# Right Pulmonary Artery Absence or Discontinuity in Ethiopian Toddler

**DOI:** 10.4314/ejhs.v30i6.24

**Published:** 2020-11

**Authors:** Henok Tadele, Etsegenet Gedlu

**Affiliations:** 1 Department of Pediatrics and Child Health, College of Health Sciences, Addis Ababa University

**Keywords:** pulmonary artery discontinuity, right pulmonary artery atresia, Ethiopia

## Abstract

**Background:**

Unilateral pulmonary artery absence or discontinuity is a rare congenital abnormality. Its reported prevalence is 1 in 150,000 adults.

**Case Presentation:**

A 22-month-toddler girl was referred from a general hospital for cardiac follow-up to our hospital after diagnosis of severe pulmonary hypertension was made. She presented with generalized body swelling, cough, fast breathing and high-grade fever of four days duration. She had repeated severe chest infections. Echocardiography and chest computed tomography revealed right pulmonary artery discontinuity. Acute care was given and chronic care was established.

**Conclusion:**

Pulmonary artery discontinuity is a rare anomaly. It should be considered in any child presenting with recurrent chest infections/pulmonary hypertension. Appropriate and timely diagnostic work up will avoid misdiagnosis.

## Introduction

Unilateral Agenesis of the Pulmonary Artery (UAPA), first reported in 1868, is a rare congenital abnormality (1). The estimated prevalence of UAPA is 1 in 150,000 adults (2). Pulmonary artery discontinuity is suggested as a favorable name for UAPA as the distal segment of the absent pulmonary artery is supplied by collaterals. The left pulmonary artery discontinuity is associated with other congenital heart diseases (CHD), while the right pulmonary artery discontinuity occurs as an isolated pathology (3).

Patients with UAPA are usually asymptomatic, and it is usually diagnosed incidentally during a workup for hemoptysis and exercise intolerance in adults. UAPA may also present in neonatal, infancy or childhood period with respiratory distress and pulmonary hypertension (4). There is no established agreement on right pulmonary artery discontinuity or UAPA management, and treatment ranges from conservative medical follow up to different types of surgeries (3,4). We report a 22-month-toddler who is diagnosed with right pulmonary artery discontinuity after she presented with recurrent chest infections. Literature review and discussion are made on this rare occurring event.

## Case Presentation

A 22-month-old toddler was referred to our hospital from the general hospital with the echocardiographic diagnosis of severe pulmonary hypertension and tiny patent ductus arteriosus. She was admitted to the referring hospital at 3, 7 and 20 months of age for the diagnosis of severe pneumonia and had received parenteral antibiotics and oxygen support. She currently presented with generalized body swelling, cough, fast breathing and high-grade fever of four days duration. She is twin B and term born child via cesarean section. Her twin brother is healthy. She is the third child for the family. There is no known maternal history of radiation exposure, infection or any intake of herbal drugs during pregnancy. Mother had antenatal care at nearby hospital, pregnancy and delivery were uneventful.

**Figure 1:**
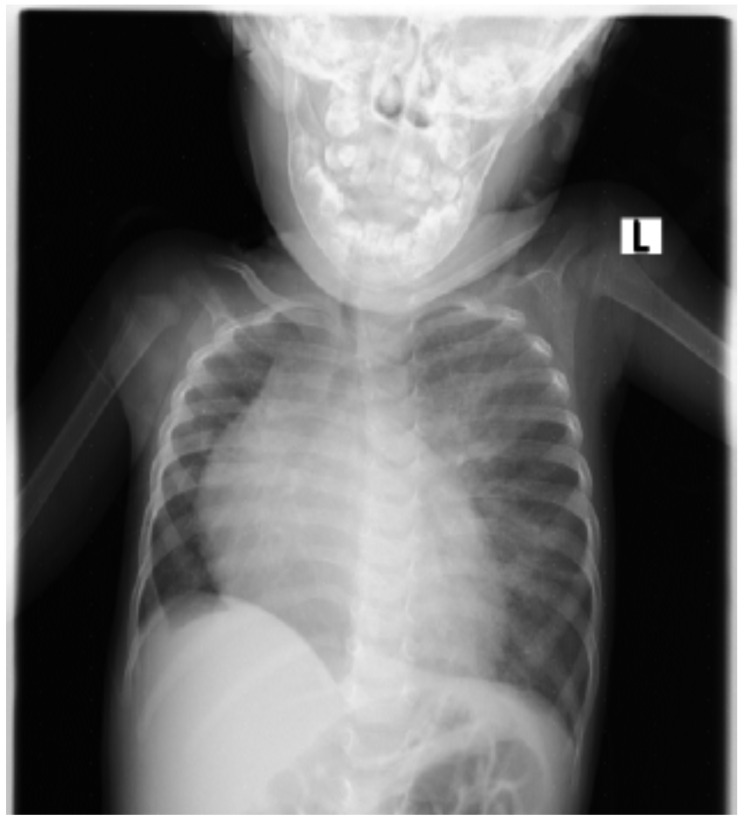
CXR of the patient showing hypoplastic right lung, trachea shifted to the right hemithorax, left upper lung zone air filled opacity, cardiomegaly, right atrial enlargement

**Figure 2:**
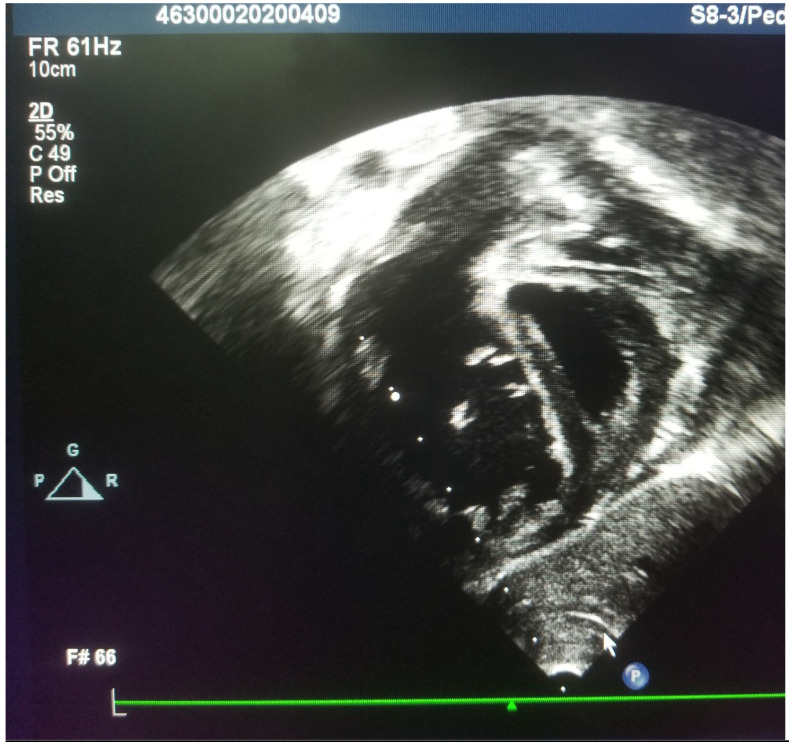
Still frame echocardiography image of subcostal short axis view at the right ventricular outflow tract displaying flat interventricular septum and D- shaped left ventricle suggesting severe pulmonary hypertension

**Figure 3:**
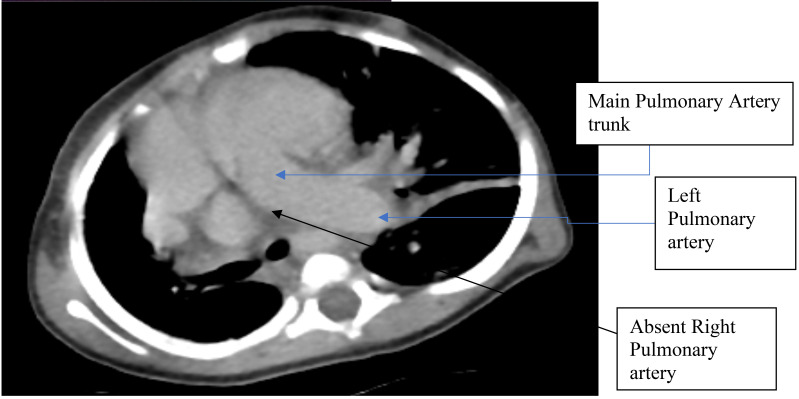
Chest CT-scan displaying absent right pulmonary artery

Physical examination showed that the child was in respiratory distress; pulse rate- 166 beats per minute, respiratory rate-66 breaths per minute, temperature- 38.5°c, SP02- 70% with atmospheric air and 92% with intranasal oxygen supplement. Her anthropometric indices based on WHO (World Health Organization) standard growth curves showed weight below -3 Z score, height between -3 and -2 Z score, and weight for height below -3 Z score. Periorbital puffiness, grunting, subcostal and intercostal retractions, bronchial breath sounds on the left posterior mid and lower two third lung field; sternal precordial budge, parasternal heave, accentuated second heart sound, grade III holosystolic murmur at the left sternal border; hepatomegaly of 11cm and grade III pedal edema were documented on examination. On investigative workup, white blood cells-7950/ml, red blood cells- 4830/ml, hematocrit-36.1% and platelets- 250,000 cells, and erythrocyte sedimentation rate-5mm/hour was documented. Serum electrolytes, wrist x-ray, renal and liver function tests were normal. Sinus rhythm, peaked P waves, and features of right ventricular hypertrophy were noted on electrocardiography. Chest x-ray (CXR) showed hypoplastic right lung, and left upper lobe air space opacity suggestive of pneumonia (Figure 1). Echocardiography showed dilated right sided cardiac chambers and main pulmonary artery, absent right pulmonary artery, severe tricuspid regurgitation-velocity of 4.3m/sec (Figure 2). Chest Computed Tomography (CT) showed right pulmonary artery atresia, ground glass opacity with septal thickening suggestive of pulmonary edema, enlarged right atrium and ventricle (See Figures 3). The child received intravenous antibiotics for the current episode of pneumonia, and started on low dose of diuretics, and digoxin. Severe acute malnutrition was also managed as per the WHO treatment protocol. The child was put on the chronic care follow-up at cardiac clinic for comprehensive care by the panel of experts.

## Discussion

Embryonically, development of pulmonary arteries is derived from the lung buds for the intrapulmonary segment and from the proximal portion of the 6^th^ aortic arch for the extrapulmonary segment. Unilateral pulmonary artery discontinuity is said to occur due to the involution of the proximal 6^th^ aortic arch. The intrapulmonary segment will fail to develop postnatally once the ductus arteriosus, an original source of blood supply, closes unless sufficient collaterals have been developed. The collaterals are reported to arise from the bronchial, subclavian or coronary arteries (4).

The clinical presentation of unilateral pulmonary artery discontinuity is diverse from asymptomatic to critical illness. Children and younger aged adults present with heart failure, recurrent chest infections, and pulmonary hypertension while other patients are diagnosed at later age when hemoptysis and exercise intolerance ensue (4). Our patient presented with recurrent chest infection and pulmonary hypertension. Unilateral pulmonary artery discontinuity results in excessive pulmonary blood flow to the contralateral lung with subsequent pathologic changes that include vasoconstriction, hypertrophy and narrowing in the small arterioles resulting in early pulmonary hypertension (4). Poor perfusion, ventilation perfusion mismatch and bronchiectasis are reported to be the common predisposing factors for recurrent chest infections (2,4).

Diagnosis of UAPA is based on history and physical examination coupled with diagnostic investigations (2,4). Diagnostic investigation includes electrocardiography (ECG), CXR, echocardiography, CT and MRI (Magnetic Resonance Imaging). ECG will be normal unless there is a pulmonary hypertension and CXR will reveal a hypoplastic lung and a shifted mediastinum towards the affected lung. Various collaterals and changes in the bronchial structure as well as pulmonary artery discontinuity will be confirmed by the CT/MRI (4).

There is no established agreement on the management of pulmonary artery discontinuity. Medical follow-up for patients who are asymptomatic is practiced and recommended. Surgical interventions are recommended for patients with hemoptysis, recurrent chest infections, or pulmonary hypertension. The surgical procedures include partial or total removal of the affected lung, and obliteration of the collaterals (4). Medical management of pulmonary hypertension needs to be instituted if surgical interventions are not under the option of care (5). Mortality rate of pulmonary artery discontinuity is 7%, younger children with pulmonary hypertension have the poor prognosis (4).

In conclusion, pulmonary artery discontinuity is a rare anomaly. Clinicians need to have a high index of suspicion for diagnosis in any child presenting with recurrent chest infections. Appropriate and timely diagnostic workup will avoid misdiagnosis.
